# Light-induced symmetry breaking for enhancing second-harmonic generation from an ultrathin plasmonic nanocavity

**DOI:** 10.1038/s41467-021-24408-x

**Published:** 2021-07-15

**Authors:** Guang-Can Li, Dangyuan Lei, Meng Qiu, Wei Jin, Sheng Lan, Anatoly V. Zayats

**Affiliations:** 1 Guangdong Provincial Key Laboratory of Nanophotonic Functional Materials and Devices, School of Information and Optoelectronic Science and Engineering, South China Normal University, Guangzhou, China; 2 Department of Materials Science and Engineering, City University of Hong Kong, Hong Kong, China; 3 Department of Applied Physics, The Hong Kong Polytechnic University, Hong Kong, China; 4 Department of Electrical Engineering, The Hong Kong Polytechnic University, Hong Kong, China; 5 Department of Physics and London Centre for Nanotechnology, King’s College London, Strand, London, UK

**Keywords:** Nanophotonics and plasmonics, Nanocavities, Nonlinear optics, Nonlinear optics

## Abstract

Efficient frequency up-conversion of coherent light at the nanoscale is highly demanded for a variety of modern photonic applications, but it remains challenging in nanophotonics. Surface second-order nonlinearity of noble metals can be significantly boosted up by plasmon-induced field enhancement, however the related far-field second-harmonic generation (SHG) may also be quenched in highly symmetric plasmonic nanostructures despite huge near-field amplification. Here, we demonstrate that the SHG from a single gold nanosphere is significantly enhanced when tightly coupled to a metal film, even in the absence of a plasmon resonance at the SH frequency. The light-induced electromagnetic asymmetry in the nanogap junction efficiently suppresses the cancelling of locally generated SHG fields and the SH emission is further amplified through preferential coupling to the bright, bonding dipolar resonance mode of the nanocavity. The far-field SHG conversion efficiency of up to $3.56\times 10^{-7}$ W^−1^ is demonstrated from a single gold nanosphere of 100 nm diameter, two orders of magnitude higher than for complex double-resonant plasmonic nanostructures. Such highly efficient SHG from a metal nanocavity also constitutes an ultrasensitive nonlinear nanoprobe to map the distribution of longitudinal vectorial light fields in nanophotonic systems.

## Abstract (web summary)

In nanoplasmonic systems symmetry can often suppress the second harmonic generation (SHG) is spite of the highly enhanced fields. Here, the authors demonstrate light-induced symmetry breaking that enables strong SHG enhancement from symmetric nanophotonic systems with certain properties.

## Introduction

Nonlinear nanophotonics has gained rapid appreciation as a route to control light with light at the nanoscale and realize many applications ranging from optical frequency conversion to imaging and biosensing as well as quantum technologies^1–4^. Both dielectric^4,5^ and metallic nanoparticles^6,7^ enable strong field enhancement inside and at the surface of a particle, respectively, and offer promising ways to enhance the nonlinear optical processes, including second-harmonic generation (SHG). High-index dielectric nanoparticles support Mie-type resonances with much larger mode volume than plasmonic nanoparticles and exhibit high SH conversion efficiencies^8,9^, compared to unstructured materials, capitalizing on a bulk second-order nonlinearity related to their non-centrosymmetric crystal lattice^10–15^. For plasmonic materials (gold, silver, etc.), the intrinsic inversion symmetry of the crystal lattice forbids the electric-dipole allowed SHG in their bulk, and the second-order nonlinear response mainly originates from the surface where the centrosymmetry is broken^16^. Such surface SHG is typically weak but much stronger localized than the SHG from dielectric nanoparticles, and very sensitive to the modification of the surface states and used for probing biochemical events, such as chemical reactions^17^, photocatalytic transformation^18^, and chemical mapping, potentially at the single-molecule level.

In the absence of the phase-matching condition important in conventional macroscale nonlinear crystals, efficient SHG in plasmonic nanostructures heavily relies on the enhancement of the fundamental field: ${I}_{2\omega }\propto {E}_{\omega }^{4}$, where ${I}_{2\omega }$ and ${E}_{\omega }$ represent the SHG intensity and the local fundamental field amplitude, respectively^19^. This strategy has been extensively implemented in various plasmonic entities to amplify the nonlinear conversion by exploiting mode resonances with stronger near-field localization of fundamental fields, such as the Fano-type resonances^20,21^. The antenna efficiency of a plasmonic nanostructure at the SH frequency also plays a significant role in the emission of the locally generated SH light^22^. Therefore, further improvement of the SHG efficiency resorted to the development of double-resonant plasmonic nanostructures that provide the fundamental field enhancement and the SH emission amplification simultaneously^23–26^, demonstrating SHG conversion efficiencies as high as $\sim\!\! 5\,\times {10}^{-10}\,{{\rm{W}}}^{-1}$. However, the high SHG conversion in these mode-matching configurations is only achievable for the near-infrared SH light for which reabsorption of the SH photons in metals is small. Due to the significant interband-transition-induced absorption loss^27^, obtaining bright nonlinear nanoscale sources in the ultraviolet-visible spectral range remains a challenge in the absence of suitable plasmonic resonances at the SH frequencies where some metals, such as Au or TiN important for practical applications, do not have plasmonic behavior at all.

The effective second-order nonlinear susceptibility of metal nanoparticles, which determines the far-field radiated SHG, strongly depends on the geometric symmetry of a nanoparticle^7,8^. At the surface of a symmetric metallic nanostructure, SHG is always associated with an anti-symmetric mode under either symmetric or anti-symmetric plasmonic excitation at the fundamental frequency. Within the electric-dipole approximation, SH emission is, therefore, available only for metallic structures with asymmetric geometries, for example, a small metallic particle with the shape deviating from a perfect sphere^28^. Beyond the electric-dipole approximation, the nonlocal, bulk electric quadrupole, and bulk magnetic dipole interactions can contribute to SHG by breaking the lattice centrosymmetry, but they are weaker than the contributions of the surface SHG^19^. Plasmonic excitations, which provide resonantly enhanced surface electric fields, can significantly amplify the SH conversion near a nanoparticle surface. Although composite plasmonic nanoantennas with a nanogap, such as a nanosphere or nanoparticle dimers or metal film-couped nanoparticles, are able to provide stronger near-fields to enhance the nonlinear polarization^29–33^, the far-field SHG efficiencies are limited by the degree of their structural symmetry^34–36^.

**Fig. 1:**
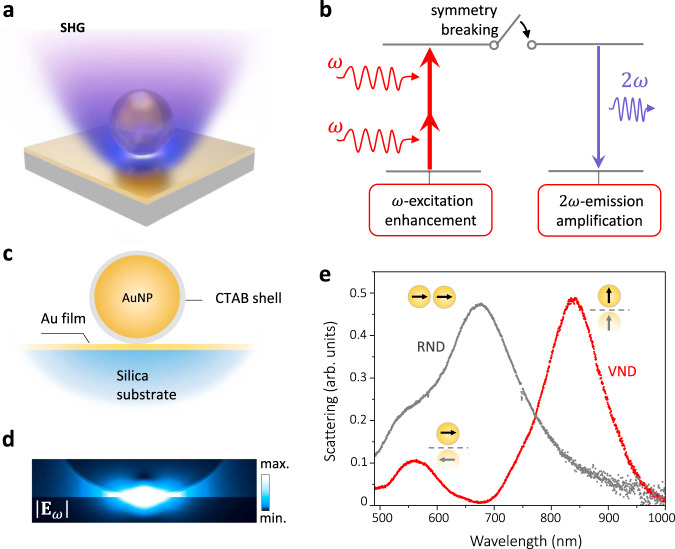
Plasmonic response of a single metal particle-on-film nanocavity and SHG enhancement mechanism. **a** Schematic of a gold particle-on-film nanocavity construct. **b** Illustration of the SHG enhancement mechanism in plasmonic nanostructures: breaking structural symmetry allows SH coupling to a bright mode of the nanostructure and acts as a switch for SH emission. **c** Schematic of a CTAB-coated gold nanoparticle (AuNP) coupled to a gold thin film on a silica substrate, equivalent to an extremely asymmetric virtual nanoparticle dimer (VND) in analogy to a symmetric real nanoparticle dimer (RND) made of two identical gold nanospheres as indicated in the inserts in (**e**). An AuNP diameter is ~100 nm and a CTAB shell thickness is ~1 nm. **d** Calculated near-field intensity distribution in the nano-construct in (**c**) at a plasmonic resonance wavelength of $850\,{\rm{nm}}$ for the electric field perpendicular to the film. **e** Dark-field scattering spectra of a gold VND (red) and an RND (gray) both with the same geometric parameters as in (**c**). Insets schematically illustrate the coupled dipole modes in the VND and RND structures corresponding to their respective resonance peaks.

Here, we demonstrate that a nanostructured plasmonic system, featuring extremely narrow gaps with an asymmetric geometry, exhibits exceptionally strong SHG in the ultraviolet-visible band. This nanostructure having a plasmonic response at a fundamental frequency consists of a single gold nanosphere closely separated from a gold film (Fig. 1a), forming a nanoparticle-on-a-mirror (NPoM) construct, which can be considered as an analog of a real nanoparticle dimer (RND) consisting of two identical nanospheres; we call it a virtual nanoparticle dimer (VND). As illustrated in Fig. 1b, such a VND has an important hidden electromagnetic asymmetry induced at the SH frequency which results in the required criteria favorable for efficient SH emission: (1) a gap plasmon resonance occurring at a fundamental frequency $\omega$, (2) electromagnetic symmetry breaking at the SH frequency in the direction normal to the film that enables a dramatic suppression of the destructive interference of locally generated SH light, and (3) related SH coupling to a bright, vertical bonding dipole mode (thus, inhibition of a non-radiative vertical antibonding dipole mode through which the SH dipoles may decay non-radiatively), resulting in a nanogap-based optical nano-antenna that efficiently radiates SH light in the far-field.

## Results

### Design of a plasmonic VND

The fabricated NPoM structure is schematically depicted in Fig. 1c, in which the nanosphere (diameter $\sim\! 100\,{\rm{nm}}$) is separated from the underlying gold film by a $\sim\! 1\,{\rm{nm}}$ thick CTAB (cetyltrimethylammonium bromide) shell coated on the particle. The plasmonic modes of such a nanostructure originate from the near-field interaction between the localized surface plasmon (LSP) of the nanoparticle and its induced image in the gold film (forming a plasmonic VND), which results in a prominent vertical bonding dipolar mode at a wavelength of $850\; {\rm{nm}}$ and a weaker, horizontal bonding dipolar mode at a shorter wavelength (Fig. 1e)^37^. The vertical dipolar mode of interest in this work features a strong electromagnetic field enhancement in the gap region with the dominant electric field component oriented in the vertical direction (Fig. 1d). On resonance, the maximum field enhancement in this extremely small gap reaches approximately 300, resulting in up to 90,000-fold enhancement of the SHG excitation rate. Using similar nanostructures, strong coupling of light emitters to the gap mode at room temperature was demonstrated, confirming a small mode volume and high electric fields^38–40^.

### Theoretical analysis of SHG in the presence of symmetry breaking

**Fig. 2:**
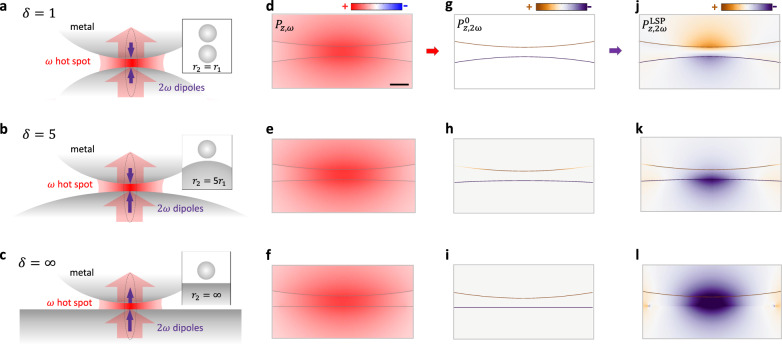
Analysis of the SH origin in gold nanodimers with different symmetry conditions. **a**–**c** Schematics of the SH origin in the nanodimers with varying degrees of structural asymmetry defined by the particle size ratio, $\delta ={r}_{2}/{r}_{1}$. Red thick arrows indicate the dipolar response to the driving field oriented along the nanogap, ${E}_{\perp }$, with the $\omega$-hot-spot forming between the nanoparticles for boosting the nonlinear source. Purple arrows represent the induced $2\omega$ dipoles responsible for the far-field SH radiation. **d**–**f** Simulated near-field polarization distributions, ${P}_{z,\omega }$, at the fundamental wavelength of 850 nm for the structures in (**a**)–(**c**) with ${r}_{1}=100$ nm and a gap $g=1$ nm. Scale bar in (**d**) is 2 nm. **g**–**i** Calculated near-field polarization distributions, ${P}_{z,2\omega }^{0}$, at the SH wavelength induced by the respective fundamental fields (Eq. (1)), which are significant only at the interface of the centrosymmetric metal. **j**–**l** SH polarization distributions resulting from the non-resonant plasmonic coupling of the surface nonlinear sources in (**g**)–(**i**) to the nanodimers. Note that spatial nonlocality and quantum tunneling are not considered in the electrodynamic calculations since they only begin to emerge in narrower metal nanogaps ($<{\;}1\;{\rm{nm}}$)^47,48^.

To understand qualitatively the mechanism of the symmetry breaking on the SHG, we analyze the nanosphere dimers of different symmetries, composed of Au nanospheres of different sizes with a nanoscale gap between them (Fig. 2). The fundamental light incident on the nanodimer from the far-field and polarized along the nanodimer axis excites the mode which can be described by in-phase bonding of 2 dipoles (the dipole can be induced in the nanosphere irrespectively of the plasmonic resonance). At the plasmonic resonance, the fundamental field oriented along the axis of the dimer experiences the strong field localization in the gap and the related field enhancement. In centrosymmetric metals, like Au, the dominating SHG originates at the metal interfaces driven by the electric field normal to the interface and can be described by the nonlinear polarization^41^

1 $${P}_{\perp }^{{\rm{S}}}(2\omega )={\chi }_{\perp ,\perp ,\perp }^{(2),{\rm{S}}}{E}_{\perp }^{2}(\omega )$$

where $P{{{\rm{S}}}\atop{\perp}}$ is the nonlinear polarization, $\chi {{\!\!\!\!\!\!(2),S}\atop{\perp ,\perp ,\perp}}$ is the dominant surface nonlinear susceptibility component, and ${E}_{\perp }$ is the normal component of the local fundamental field at the metal interface. Therefore, the SH response of the nanodimers mainly originates from the nonlinear polarization induced at the interfaces of the metal at the gap junctions, with the field at each interface directed outwards the metal. In the case of symmetric nanoparticles (Fig. 2a), the SH dipoles are equal but oppositely oriented (antibonding). Therefore, despite the strong SHG generated in the near-field due to the fundamental field enhancement (Fig. 2d), the far-field radiated SHG is quenched due to the destructive interference of antiparallel dipoles (Fig. 2g) through preferential coupling to the dark, antibonding plasmon mode of the RND (Fig. 2j), and consequently the energy is dissipated non-radiatively due to the losses in metal. This is confirmed by the numerical simulations (Fig. 2d, g, j) based on the phenomenological free-electron model (Supplementary Note 1).

In the case of an asymmetric nanodimer (Fig. 2b), while the nanostructure still produces the antibonding type SH dipole distribution, the imbalance between them results in some far-field SHG, which potentially can be enhanced if the plasmonic resonance of the nanostructure can be excited in the dimer. These 2 scenarios in Fig. 2a, b correspond to the RND configuration. The situation becomes dramatically different for the VND configuration (Fig. 2c). While the nonlinear polarization distributions are still antisymmetric as expected (Fig. 2i), the resulting dipolar moment has an in-phase polarization distribution across the gap by exciting the bright, bonding plasmon mode of the VND (Fig. 2l), thus rendering a radiative mode for the SH emission. This mode character is exclusive to the VND construct because the SH-dipole-image-mediated coupling between the particle and the film in the VND forbids the formation of an antibonding-type mode^37^. In sharp contrast to the SH “cold spot” observed in the nanogap of the RNDs (Fig. 2j, k), an intense SH “hot spot” observed in the VND gap indicates a strong and highly localized nonlinear source. This effect does not require plasmonic resonance at the SH frequency, which might be difficult to achieve in the UV, and is based on the bonding of the induced SH dipoles.

The electromagnetic asymmetry induced at the SH frequency in the plasmonic VND system occurs through two steps: (1) the local SHG polarization (${P}_{z,2\omega }^{0}$ in Fig. 2g–i as described by Eq. (1)) has an asymmetric distribution on the surface of the asymmetric RND and the VND and (2) The SH asymmetry is further increased through the preferential coupling of the SH light to a bright, bonding plasmon mode of the VND, giving rise to the global SHG polarization (${P}_{z,2\omega }^{LSP}$ in Fig. 2j–l). The plasmonic excitations at a fundamental frequency in the symmetric and asymmetric RNDs (Fig. 2a–c) are all bonding-dipolar plasmonic modes (Fig. 2d–f). According to Eq. (1), the SHG polarization direction in each of the three systems is determined by the normal to the interfaces (i.e., directed outwards the metal), which makes the local SHG polarization of the three systems all anti-bonding-type. However, the significant difference among these anti-bonding modes is that the nonlinear polarization on either the bigger gold nanosphere and the gold film is always stronger than that for the smaller nanosphere due to the larger surface area (Fig. 2g–i), and the imbalance between them may be further amplified through the excitation of the bonding plasmonic mode of the VND structure at the SH frequency.

### Experimental observation of SHG from VND

**Fig. 3:**
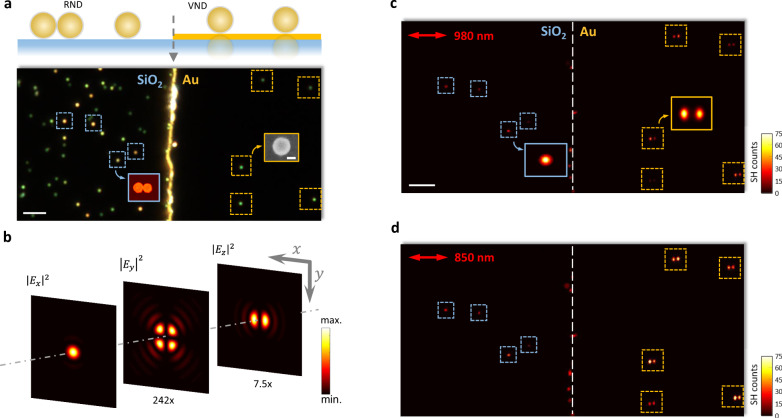
SHG confocal mapping for RND and VND nanostructures. **a** Dark-field image of two separate areas containing gold nanospheres dispersed on silica and gold films, respectively. On a silica substrate, the bright yellow spots correspond to the RND structures (with $\delta$ typically ranging from 1.0 to 1.26 as confirmed by the AFM imaging), while the green spots correspond to gold nanosphere monomers. On a gold substrate, 5 VNDs appear as green spots. Inset shows the SEM micrograph of a single VND. The scale bar is 50 nm. **b** Calculated intensity distributions for the three field components of a highly focused laser beam at the focal plane of a 100× objective (numerical aperture 0.95). The incident beam is polarized along the $x$ direction and has a wavelength of 850 nm, corresponding to the SHG experimental conditions. **c**, **d** SHG confocal images of RNDs (left) and VNDs (right) under off- ((**c**), 980 nm) and on-resonance ((**d**), 850 nm) fs-laser excitation. The time duration of the laser beam dwelling at each pixel is 18 μs. The double-headed red arrows indicate the polarization direction of the fundamental light. Insets in (**c**) show the images for the RNDs and VNDs scanned with higher resolution and longer pixel dwelling time (72 μs). The color bars indicate the SH intensity in photon counts per μs. Only several RNDs are visible in the SHG images with the axes oriented along with the fundamental light polarization (see Supplementary Note 5). Scale bar in (**a**), (**c**) is 5 μm.

To validate the advantage of the VND configuration for SHG superior to a RND, we performed nonlinear confocal scanning measurements on both the single gold NPoMs and the nanodimers (see Methods and Supplementary Note 2). This allows direct comparison of the SHG efficiency from the VNDs in the NPoM configuration providing the largest asymmetry, $\delta =\infty$, and from the RNDs with the smallest asymmetry, $\delta =1$. To realize these configurations experimentally, gold nanospheres were dispersed on a silica substrate with a half area covered by a gold thin film (see Methods). In this way, self-assembled RNDs on the silica substrate can always be found and optically identified by their bright yellow colors in the dark-field images (Fig. 3a)^30^. SHG measurements were performed in confocal configuration with a pulsed femtosecond laser beam with a Gaussian profile as the excitation source. To provide sufficient longitudinal field intensity to trigger the nonlinear process in the VND structure, the laser beam was tightly focused by a dry objective with a high numerical aperture (NA = 0.95), which produces the focal field distributions as presented in Fig. 3b (see Supplementary Note 3 for details).

As seen from Fig. 3c, even under the off-resonance excitation condition (980 nm), almost all the VND structures are visible in the SHG image, whereas only a few RNDs with dimer axis oriented along the fundamental light polarization can be identified due to a weaker SHG intensity (blue boxes). In the latter case, a silica substrate, which has negligible SHG itself, induces symmetry breaking. The observed SH intensity variation between the monitored RNDs may be ascribed to the different axis orientation with respect to the excitation light polarization and slight asymmetry ($\delta \,\ne\, 1$) due to size dispersion of the nanospheres as seen from the AFM image (Fig. 3a, inset). The SH confocal image of the single RNDs appears as a solid spot (Fig. 3c, inset) while that of the single VNDs shows a two-lobe-shaped pattern, characteristic of the ${E}_{z}$ field distribution needed to excite the bonding dipolar resonance mode of the NPoM (Fig. 3b). This confirms that the SHG process in the RNDs is driven by the dominant transverse field component (${E}_{x}$), while in the single VNDs only by the longitudinal field component (${E}_{z}$). These two field components correspond to the directions along which symmetry breaking is required for the dipole-allowed SH emission in the dimer nanostructures. Moreover, the ${E}_{x}$ field component responsible for the SH emission from RNDs has more than 7 times larger peak intensity than that of the longitudinal field ${E}_{z}$ (Fig. 3b) but renders significantly weaker SH radiation from the RNDs than that from the ${E}_{z}$ driven VNDs.

The SH intensity contrast between the RNDs and VNDs is even more pronounced when the fundamental wavelength is tuned to match the gap plasmon resonance (~850 nm, Fig. 1e) of the individual VND structures (Fig. 3d). This resonant excitation enhances the local field intensities in the nanogap region of the single VNDs by $\sim\! 10$ times compared to the 980 nm excitation, which is corroborated by the comparison between the scattering spectrum and the SHG excitation spectrum of the single VNDs (Supplementary Fig. 8). Under these conditions, a direct comparison of the SH intensities from a single VND and a single RND reveals $\sim\! 13.5$-fold stronger SHG from VNDs. This direct evaluation is, however, significantly underestimated because the ${E}_{z}$ field intensity is significantly smaller than that of the ${E}_{x}$ component (Supplementary Note 3).

After careful evaluation of the losses and the collection efficiency in the detection path of the experimental setup, we can determine an SH conversion efficiency $\eta =P(2\omega )/{P}^{2}\left(\omega \right)$ to be approximately $3.56\,\times\, {10}^{-7}$ W^−1^ for a single VND, based on the statistic measurements of 271 VNDs (Supplementary Note 8). Each VND on average emits $\sim\! 1.49\,\times {10}^{9}$ photons per second, which exceeds that of doubly-resonant and broadband plasmonic nanoantennas by more than two orders in magnitude^26,42^. Even though the SHG emission spectrally overlaps with the interband transitions of gold, which introduces significant losses, the efficiently emitted SHG radiation overcomes the reabsorption of SH photons.

**Fig. 4:**
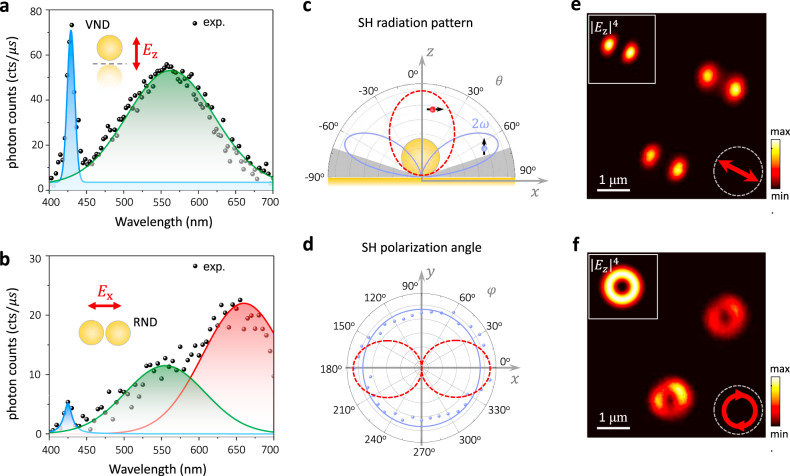
Characterization of SHG from individual nanosphere dimers. **a**, **b** Emission spectra of the VND (**a**) and the RND (**b**) labeled by solid squares in Fig. 3c. The solid lines are fittings to the SHG (blue) and TPL (green and red) spectra, respectively. The two TPL peaks in (**b**) are related to two resonances observed in the scattering spectrum (Fig. 1e). **c** Calculated SHG radiation pattern of a single VND (blue) and a comparison to the SHG radiation pattern of a horizontal electric dipole above a silica substrate, emulating an RND (red). **d** Measured (blue dots) and simulated (blue line) polar plots of the polarization of the SHG light from an individual VND. The red curve represents the calculated SHG polarization orientation for an RND emulated with an electric dipole. The measured pattern is slightly stretched along $x$-axis due to the polarization response of the experimental setup. **e**, **f** Comparison between the SHG confocal images of the VND scanned using linearly (**e**) and circularly (**f**) polarized fundamental light. Insets show the calculated driving longitudinal field |*E*_z_|^4^ in the focal plane. Scale bar in (**e**), (**f**) is 1 μm.

Figure 4a, b shows the emission spectra of individual RNDs and VDNs, respectively. A prominent SH emission peak is observed for the single VNDs, accompanied by a broad two-photon luminescence (TPL) peak with comparable intensity. The TPL peak occurs at the anti-bonding dipolar resonance wavelength $\sim\! 550\; {\rm{nm}}$ (Fig. 1e), which provides a doubly-resonant enhancement mechanism for the TPL emission in the single VNDs. It is important to point out that the TPL process in plasmonic metals is not symmetry-forbidden^43^. The comparable SH intensity with respect to that of the TPL emission indicates a significant relaxation of the symmetry restriction in the dipolar SH radiation from single VNDs. In contrast, the SH emission from single RNDs shows a much smaller SHG intensity compared to that of the accompanying TPL (Fig. 4b). This is in line with the prediction considering the limited asymmetry degrees of a single RND structure.

Based on the calculated nonlinear near-field polarization distributions in Fig. 2, the near-to-far field transformation (NFFT) is performed to understand the far-field SH radiation from single VNDs and RNDs. Indeed, the calculation results indicate a dipolar character of the emission from VNDs with polarization moment aligned vertically (Fig. 4c), being of a similar nature to that of the bonding dipole mode at the fundamental wavelength^44^. Such a dipolar emission character has rotational symmetry around the VND axis, in line with the measured isotropic polarization of the SH radiation from the single VNDs (Fig. 4d). Under the circularly polarized excitation, which has a donut-like longitudinal field distribution, the SHG image of the VND has a radial intensity distribution, revealing an opportunity for longitudinal field mapping (Fig. 4f).

## Discussion

Plasmonic nanoparticle-on-a-film structures provide, in addition to the field enhancement, strong asymmetry of the induced second-order polarization, resulting in the efficient emission of the SH radiation due to formation of a bright VND dipole at a SH frequency. The inhibition of a nonradiative antibonding plasmon mode in this nanostructure enables increased out-coupling efficiency of SHG via coupling of the SH photons generated in the near-field to the radiative bonding dipole mode, thus rendering a bright nonlinear light source at the nanoscale. For the same excitation, the SH intensity from a single VND is more than 13 times higher than the SHG from an RND with weak asymmetry, achieving an SH conversion efficiency of $3.56 \times {10}^{-7}$ W^−1^ corresponding to the SH photon emission rate $\sim\! 1.49 \times {10}^{9}$ s^−1^. Therefore, the described design strategy outperforms doubly-resonant and broadband plasmonic nanoantennas by more than two orders in magnitude. Since the nonlinear response of this plasmonic nanostructure is dominantly driven by a longitudinal field component, the single metal nanoparticles on a film can act as a nanoprobe for mapping longitudinal electromagnetic fields with high spatial resolution. For the same reason, higher second harmonic intensity in these extremely asymmetric nanodimers can be expected with optimally structured light, for example, radially polarized or other cylindrical vector beams that produce a large longitudinal field in the focal plane^45^. Normalized to the same excitation field, the radiated SH intensity from a single VND would exceed that from RND by more than 650. We also anticipate that the design strategy adopted here can be generalized to other nonlinear nanomaterials featuring axis or inversion symmetry for highly efficient light conversion without the need of plasmonic resonances at the SHG frequencies, important for UV SHG generation, and ultimately, achieving the nonlinear nanophotonic devices such as bright nanoscale light source, including for quantum technologies, imaging and sensing of biological objects, and ultrafast on-chip optical switches.

## Methods

### Sample fabrication

50-nm-thick gold films are deposited on silica substrates using thermal evaporation. The deposition process is conducted in a high-vacuum chamber ($\sim\!\! 3\times {10}^{-7}$ Torr., Nexdep, Angstrom Engineering Inc.). The averaged surface roughness is $\sim\! 0.5\;{\rm{nm}}$ as measured by an atomic force microscope (AFM, Nanoscope V, Veeco). In each silica-supported gold film, part of the gold film is mechanically erased, leaving the underlying silica substrate exposed. Gold colloidal nanoparticles with a nominal diameter $100\pm 8\;{\rm{nm}}$ (Nanoseedz Inc., Hong Kong) are rinsed and diluted in deionized water to reach a relatively low concentration, after which a droplet of the as-prepared nanoparticles is directly cast onto the previously prepared Au-silica substrate and left dry in air. The distances between individual nanoparticles on the gold film are typically larger than 5 μm and larger than 2 μm on a silica substrate, which allows for optical characterization of individual nanoparticles. On a silica substrate, due to the self-assembly processes, nanoparticle dimers are formed in addition to the dominated monomers. The colloidal gold nanoparticles with CTAB surfactant used in this study were synthesized by a well-documented seed-mediated growth method^46^. With this method, the CTAB surfactant forms a single bilayer configuration at the surface of the nanoparticles, and its thickness ($\sim\!\! 1$ nm) determines the distance between the nanoparticles and the underlying gold film.

### Optical characterization and SHG spectroscopy

The plasmonic resonances of individual gold nanoparticles and dimers are optically characterized with a reflective-type dark-field microscope equipped with a spectrometer system. By focusing the incident white light with a 100× dark-field objective, a taper-shaped hollow beam with high convergence angle (up to 69°) is produced to illuminate the nanoparticles. The scattered light from the individual nanostructures is collected by the same objective with a smaller collection angle (53°) and then delivered to either the spectrometer for spectral analysis or the camera for wide-field imaging.

The SHG spectroscopy of the plasmonic nanostructures is implemented on a commercial laser scanning confocal microscope system (Leica, TCS SP5) combined with a Ti: sapphire femtosecond laser (Spectra-physics, Mai Tai HP). The laser beam (pulse width 120 fs, repetition rate 80 MHz) is tightly focused on the sample with a high NA (0.95) objective. The emitted second harmonic photons from the plasmonic nanostructures are collected by the same objective in an epi-reflection configuration and then sent to high-sensitivity hybrid detectors (HyDs) for photon counting. The confocal image reconstruction within the SH band and spectral analysis are performed by a prism-based light dispersion module inserted in the detection path. To minimize the laser damage, the laser power used is $< 1\; {\rm{mW}},$ and the time duration of laser spot dwelling at each pixel is extremely short, typically $< 20$ μs. Although previous studies reported the morphology modifications that occurred in the metal nanogaps when irradiated by continuous-wave lasers, here we observe no optical signatures of such unfavorable effects during the extremely short measurement time (Supplementary Note 4). Control experiments on gold nanospheres with and without CTAB coating show that their far-field SH response originates predominantly from gold nanoparticles rather than the CTAB coating (Supplementary Note 9).

### Numerical FEM calculations

The SH near-field polarization fields of the nanodimer structures were simulated numerically using a commercial finite-element solver (COMSOL Multiphysics) in the frequency domain. A perturbative approach within the undepleted-pump approximation (i.e., the generated harmonic field does not couple back to the fundamental field) was employed to run the simulations. The SH responses from plasmonic metals originate from the free-electron currents induced by both bulk and surface effects, but here we only consider the dominant ones normal to the metal surface in the simulations. The far-field SH radiation patterns of individual plasmonic nanostructures were derived by performing a near-to-far-field transformation of the simulated fields at the SH wavelength (Supplementary Note 1).

## Supplementary information

Supplementary Information

## Data Availability

The data that support the findings of this study are available from the corresponding author upon reasonable request.
