# Supplementary material for: Light-induced symmetry breaking for enhancing second-harmonic generation from an ultrathin plasmonic nanocavity
Source: Nat Commun. 2021 Jul 15;12:4326. doi: 10.1038/s41467-021-24408-x (PMC8282679; doi:10.1038/s41467-021-24408-x)
Supplement: Supplementary file 1 — Supplementary Information [file 41467_2021_24408_MOESM1_ESM.pdf]

Supplementary Information

Light-induced symmetry breaking for enhancing second-harmonic generation from an ultrathin plasmonic nanocavity

Guang-Can Li[1,2,3], Dangyuan Lei[2,3,*], Meng Qiu[4], Wei Jin[4], Sheng Lan[1], Anatoly V. Zayats[5,$]

[1] Guangdong Provincial Key Laboratory of Nanophotonic Functional Materials and Devices, School of Information and Optoelectronic Science and Engineering, South China Normal University, 510006 Guangzhou, China.

[2] Department of Materials Science and Engineering, City University of Hong Kong, Hong Kong, China.

[3] Department of Applied physics, The Hong Kong Polytechnic University, Hong Kong, China.

[4] Department of Electrical Engineering, The Hong Kong Polytechnic University, Hong Kong, China.

[5] Department of Physics and London Centre for Nanotechnology, King's College London, Strand, London WC2R 2LS, UK.

*e-mail: dangylei@cityu.edu.hk

$e-mail: a.zayats@kcl.ac.uk

Content

1. Numerical methods for SHG simulations

2. Experimental setup for nonlinear optical imaging and spectroscopy

3. Calculations of the field distributions in the focal plane

4. Evaluation of laser damage to the metal film-coupled nanoparticles

5. Optical identification of the RNDs and determination of their orientations

6. Excitation power dependence of the SHG and TPL

7. Excitation spectrum of the SHG from single VNDs

8. Evaluation of the second harmonic conversion efficiency

9. Determination of the SH origin of CTAB-coated gold nanoparticles

Supplementary References

1. Numerical methods for SHG simulations

In the nonlinear simulations, we adopted the perturbative approach within the undepleted-pump approximation, which is justified by the fact that the SH field intensity generated by a plasmonic nanostructure is always orders of magnitude weaker than that of the excitation field. Under this approximation, the SH field does not couple back to the pump field and, therefore, the nonlinear process can be decomposed into two linear scattering processes: firstly, the fundamental fields were solved with the finite element solver in the frequency domain and the calculated fields were used to find the SH polarization source currents that are responsible for the far-field SH emission. Although both the free and bound electrons in metals can give rise to the second-order nonlinear process, it has been proven that only the former is responsible for the dominant SH response in the visible and near-infrared spectral range[1]. The free-electrons-related SH polarization currents can be further decomposed into the volume and surface contributions. Here, we only consider the second-harmonic polarization which is normal to the metal surface for it plays the dominant role in the SHG[2,3]. In this case, the SH source currents based on the free-electron hydrodynamic model can be found as[3]

$$\hat{\mathbf{n}} \cdot \mathbf{J}_{\text{surf}} = i \frac{n_0 e^3}{2m_*^2} \frac{3+\varepsilon_{\text{EF}}(\omega)}{(\omega+i\gamma)^2(2\omega+i\gamma)} E_\perp^2(\omega) \tag{1}$$

where $\hat{\mathbf{n}}$ is the unit vector normal to the metal surface, $\mathbf{J}_{\text{surf}}$ is the surface current, n_0 is the free-electron density in gold, m_* represents the effective electron mass, e is the elementary charge, γ is the electron gas collision frequency in gold, ε_{EF} is the bulk gold permittivity at the fundamental wavelength (taken from Ref. 16 in the main text), and $E_\perp(\omega)$ is the normal component of the local fundamental field at the metal surface. Due to the limited penetration of the electromagnetic fields into the bulk gold, the induced nonlinear polarization occurs mainly in the sub-nanometer-thick surface layer. Considering the rotational symmetry of a VND, the simulation configuration can be simplified into a two-dimensional frame for which the meshing for the surface layer, where the SH source is located, can be scaled down to the level of 0.1 Å (Supplementary Fig. 1). For the RND on silica, we neglect potential substrate effects and, therefore, the substrate is not included in the simulation. In addition, the nonlocal[4] and quantum tunneling[5] effects are not included here since these effects occur within metal-sandwiched vacuum gaps typically with sizes below 1 nm[6,7].

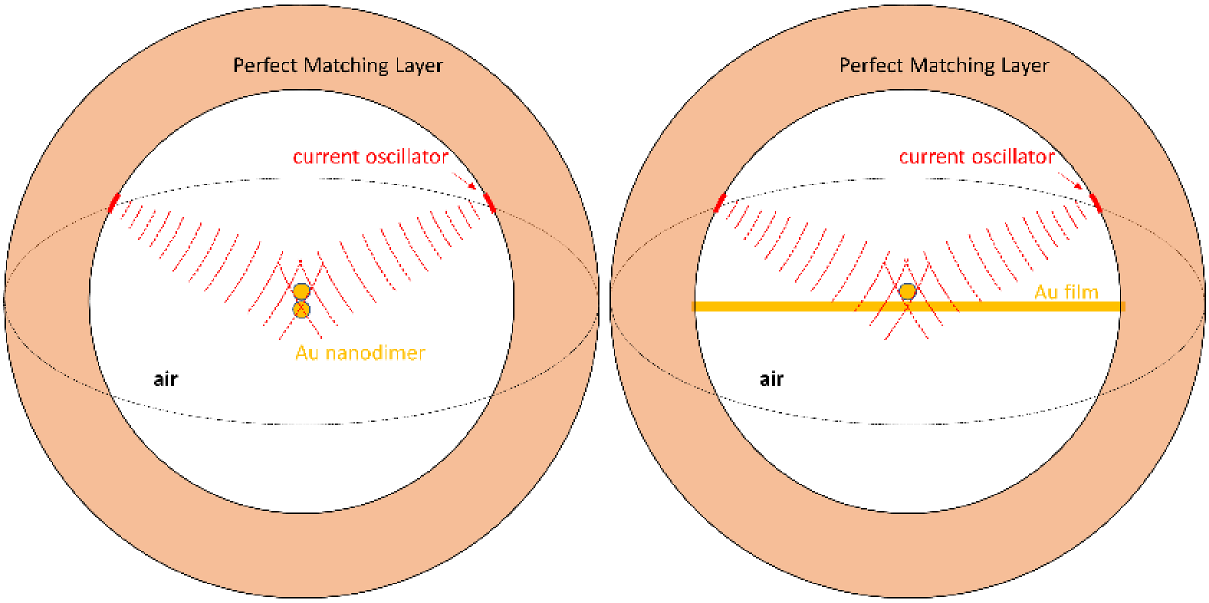

Supplementary Figure 1. Simulation domains for a single RND (left) and VND (right). The excitation source is modeled by the current oscillator with the oscillation direction tangential to the inner surface of the perfectly matching layer. Such excitation configuration can preferentially excite the gap plasmon modes of the nanodimers.

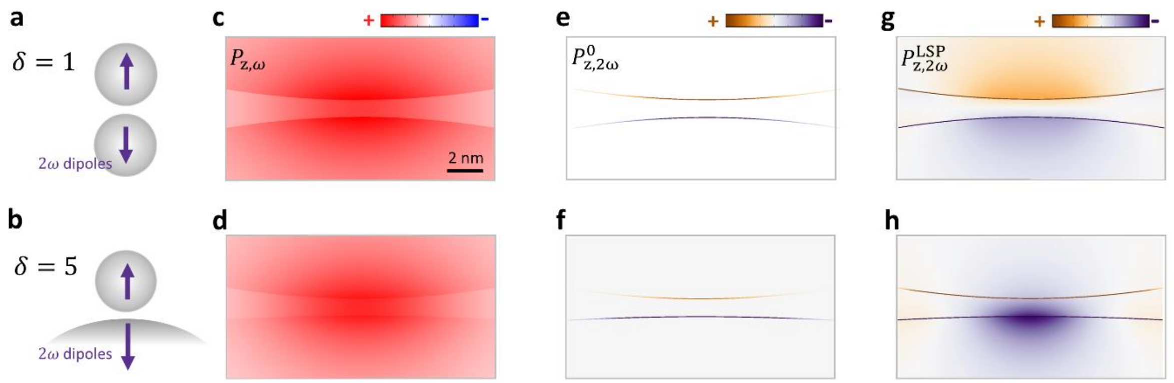

Supplementary Figure 2. Analysis of the SH origin in a single RND upon resonant excitation of the gap plasmon modes. a, b Schematics of the SH origin in the nanodimers with varied degree of structural asymmetry. **c, d** Simulated near-field polarization distributions, $P_{z,\omega}$, at the gap plasmon resonance wavelengths (680 nm for the $\delta = 1$ case and 830 nm for the $\delta = 5$ case) for the structures in (**a**, **b**) with $r_1 = 100$ nm and a gap distance $g = 1$ nm. **e, f** Calculated near-field polarization distributions, $P^0_{z,2\omega}$, at the SH wavelength induced by the respective fundamental fields. **g-h** SH polarization distributions resulting from the resonant plasmonic coupling of the surface nonlinear sources in (**e**, **f**) to the nanodimers.

2. Experimental setup for nonlinear optical imaging and spectroscopy

As illustrated in Supplementary Fig. 3, a Ti:sapphire femtosecond laser (Spectra-physics, Mai Tai HP) with a tunable wavelength range of $690 - 1040$ nm delivers laser pulses (pulse duration $\sim$120 fs) into an inverted microscope (Leica, TS SP5). A half-wave and quarter-wave plate (HWP &QWP) combination inserted into the beam path controls the polarization state of the laser beam. A $100\times$ dry objective (HCX PL APO) with $\mathrm{NA} = 0.95$ is used to focus the incident beam into a diffraction-limited spot. The SHG signal from metal nanoparticles in the focal plane is collected by the same objective, and then reflected into the detection path by a short-pass dichroic mirror which removes the pump laser and allows the SHG signal to pass. A high-sensitivity hybrid detector (HyD) which is capable of resolving light intensities at a few photon level is used to detect the SH light. The filter tuning module before the detector optics can be dynamically controlled to select the detection spectral range. The emission spectra are acquired by sliding a 10-nm-wide detection window across the broad visible band with a 5 nm step.

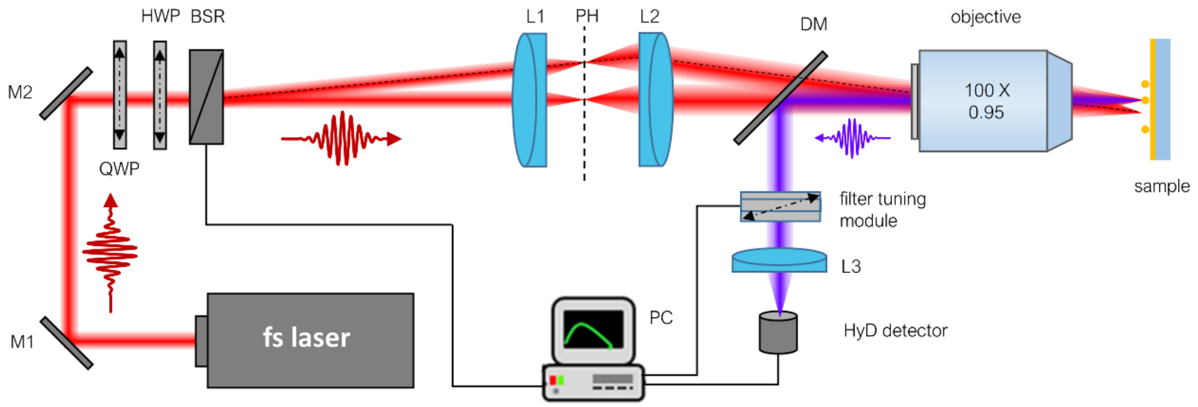

Supplementary Figure 3. Experimental setup for nonlinear optical imaging and spectroscopy: fs laser - femtosecond laser; M1, M2 - mirror 1 and mirror 2; HWP - half wave plate; BSR - beam scanning resonator; L1, L2 - lens 1 and lens 2; DM - dichroic mirror; PH - pinhole; HyD detector – hybrid detector. Filter tuning module is used to spectrally control the detection window for imaging and resolve the emission spectrum.

3. Calculation of field distributions in the focal plane

In the confocal imaging experiments, the mapped SHG intensity patterns of individual nanostructures are strongly dependent on the excitation field distributions in the focal plane. Based on the vectorial diffraction theory, the focal fields distribution of a linearly ($\mathbf{E}_{\mathrm{L}}$) or circularly ($\mathbf{E}_{\mathrm{cir.}}$) polarized beam can be respectively calculated by[8,9]

$$\mathbf{E}_{\mathrm{L}} = \begin{bmatrix} E_{\mathrm{x}} \\ E_{\mathrm{y}} \\ E_{\mathrm{z}} \end{bmatrix} = \begin{bmatrix} -i\left(I_0 + I_2\cos(2\varphi)\right) \\ -iI_2\sin(2\varphi) \\ -2I_1\cos(\varphi) \end{bmatrix} \quad (2)$$

$$\mathbf{E}_{\text{cir.}} = \begin{bmatrix} E_\text{x} \\ E_\text{y} \\ E_\text{z} \end{bmatrix} = \begin{bmatrix} -i\left[I_0 + I_2 e^{i2\varphi}\right] \\ I_0 - I_2 e^{i2\varphi} \\ -2I_1 e^{i\varphi} \end{bmatrix} \tag{3}$$

with

$$I_0(\rho) = \int_0^{\text{ksin}\alpha} F_0\,(\kappa) J_0(\kappa\rho) e^{i\text{z}\sqrt{\text{k}^2-\kappa^2}} \text{d}\kappa \tag{4}$$

$$I_1(\rho) = \int_0^{\text{ksin}\alpha} F_1\,(\kappa) J_1(\kappa\rho) e^{i\text{z}\sqrt{\text{k}^2-\kappa^2}} \text{d}\kappa \tag{5}$$

$$I_2(\rho) = \int_0^{\text{ksin}\alpha} F_2\,(\kappa) J_2(\kappa\rho) e^{i\text{z}\sqrt{\text{k}^2-\kappa^2}} \text{d}\kappa \tag{6}$$

where α is the maximum focusing angle determined by the objective numerical aperture via $\text{NA} = n\sin(\alpha)$, $\kappa = \text{ksin}\theta$ is the spatial frequency with θ representing the convergence angle between the rays in the focal image and the optical axis, and ρ, φ, z denote coordinates in the focal plane. The exact forms of F_i $(i = 0, 1, 2)$ can be found in Ref. 10. The power weight of the three field components in the focal plane can be roughly estimated by the ratio $\eta_i = \frac{P_i}{P} = \frac{\iint E_i^2 ds}{\iint (E_x^2 + E_y^2 + E_z^2) ds}, i = x, y, z$ (see Supplementary Fig. 4a). In the experiment, the objective has a NA of 0.95. For a linearly polarized (here, *x*-polarized) Gaussian beam, the power weight of each field component in the focal plane can be numerically calculated (Supplementary Table 1). Table 1 also shows the results calculated for a radially polarized (RP) vector beam, which shows a dominant longitudinal field component (E_z).

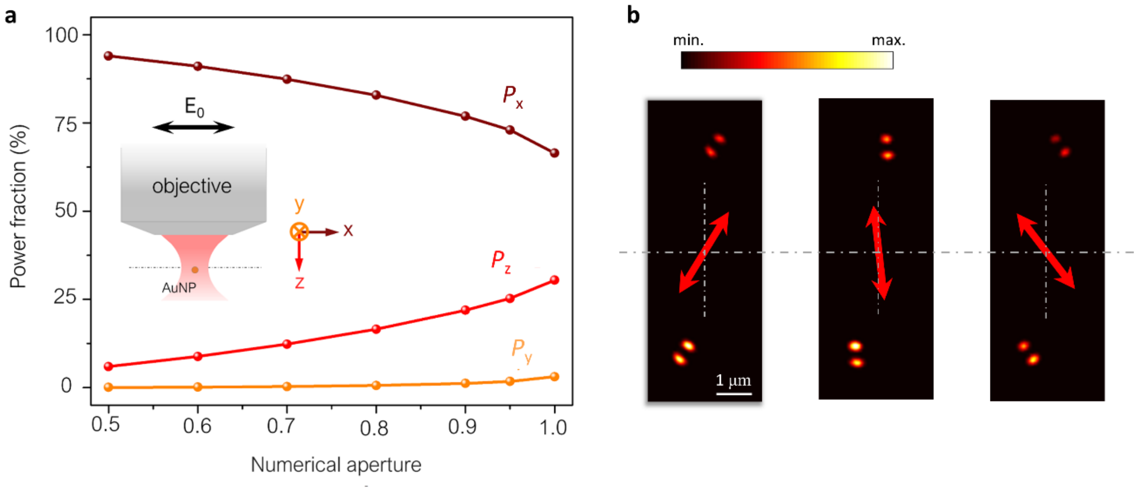

Supplementary Figure 4. a Calculated power fraction of the field components of a *x*-polarized Gaussian laser beam in the focal plane of an objective, exhibiting a strong dependence on the objective numerical aperture. **b** SHG confocal maps of the single VNDs measured using the focused fundamental light with different linear polarization directions indicated by double-headed arrows. The detection band for the SHG confocal mapping is 420-430 nm.

Supplementary Table 1. Power weight of the constituent field components calculated for tightly focused laser beams (NA=0.95).

Beam type	η_x	η_y	η_z
x- polarized Gaussian	81.97%	0.85%	17.17%
RP	24.67%	24.67%	50.66%

It should be pointed out that for the comparison of the SHG intensities of single RNDs and VNDs, the rescaling of the as-measured SHG intensities is needed due to the different intensities of the fundamental light of different polarization components. The SHG from RNDs is driven by the x-field component, while that from the VNDs by the z-field component (Figs. 3c-d). These two field components have different intensities in the focal plane (Fig. 3b). The peak intensity of the x-field component in the focal plane is estimated to be ~7.5 times that of the z-component. Since $I_{\mathrm{SHG}} \propto E^4$, this pump intensity difference would give rise to an apparent >50-fold stronger SHG signal from the RNDs. In experiment, this has to be taken into account when comparing SHG conversion efficiencies of different nanostructures.

4. Evaluation of laser damage to the metal film-coupled nanoparticles

To minimize the laser-induced damage to the gold nanoparticles, during the scanning process, the focused laser spot dwells at each pixel for an extremely short time, typically < 20 µs. The

average pump power is set to be < 1 mW at the focal plane. The possible laser damage is evaluated using multiple approaches. The optical dark-field images captured for single nanoparticles would show significantly different spectra and scattering patterns before and after the laser damage occurs. As indicated in Supplementary Fig. 5b, the scattering pattern of single gold nanoparticles on gold film transforms from a doughnut-shaped profile to a solid spot when the laser-induced damage breaks structural symmetry of the nanoparticles. This is also confirmed by the SEM micrographs (the insets in Supplementary Fig. 5b). However, possible minute changes in the particle-film gaps cannot be resolved with these imaging approaches. Since the gap plasmon resonance of a single metal nanoparticle-on-film structure is sensitive to the gap morphology and distance, we rely on the comparison of the measured scattering spectra of single nanostructures before and after the laser irradiation. For the laser irradiation power ~ 0.6 mW, most of the nanoparticles on gold film show scattering spectra as in Supplementary Fig. 5c, which indicates negligible particle-film gap variation. Moreover, the SHG signal is extremely sensitive to the minute structural geometry modification, and the SHG confocal imaging can be employed to monitor the structural stability during laser scanning. As indicated by the well-defined SHG patterns in Supplementary Fig. 9d, the individual nanoparticles on gold film exhibit excellent morphology stability during the scanning process with < 8 ms dwelling time.

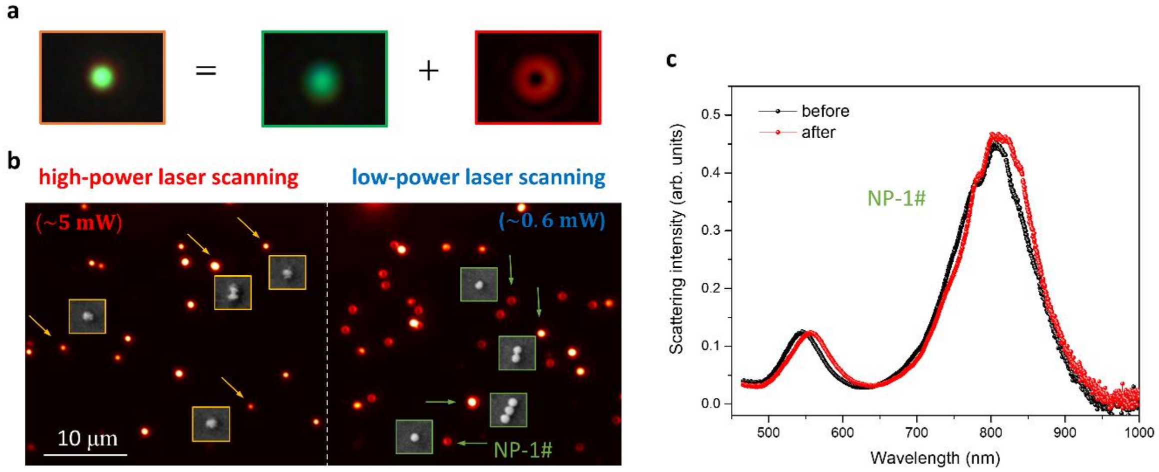

Supplementary Figure 5. a The decomposition of the dark-field scattering pattern of a single VND, corresponding to the 2 resonances in the scattering spectrum. The far-field radiation pattern of the gap plasmon mode of the VND (the bonding dipole mode) can be visualized in the red CCD channel (corresponding to the detection band of 600 - 900 nm). **b** Comparison between the dark-field images of two different sample regions scanned with high (~5 mW, left) and low (~0.6 mW, right) laser power. The images are extracted from the red channel of the CCD. The individual metal film-coupled nanoparticles in the undamaged region (right) show clear doughnut-shaped radiation patterns, while their counterparts in the damaged region appear as solid dots. **c** Representative scattering spectra of a single VND (as labeled in (**b**), with diameter ~ 100 nm) measured before and after the laser scanning with the illumination intensity and dwell time used for the SHG measurements. The laser power is ~ 0.6 mW as measured at the back focal plane of the focusing objective.

5. Optical identification of the RNDs and determination of their orientations

The individual nanosphere monomers and dimers on a silica substrate show significantly different scattering responses, which are manifested in both their far-field scattering patterns and spectra (Supplementary Fig. 6). These distinct properties can thus be employed to identify the nanoparticle dimers from the monomers and to determine their in-plane orientations with the polarization-resolved dark-field microscopy. Using this noninvasive optical method, we identify that the observed silica-supported-particles in the SHG confocal image in Supplementary Fig. 6b are nanodimers with axis orientations preferentially aligned along the dominant *x*-field component.

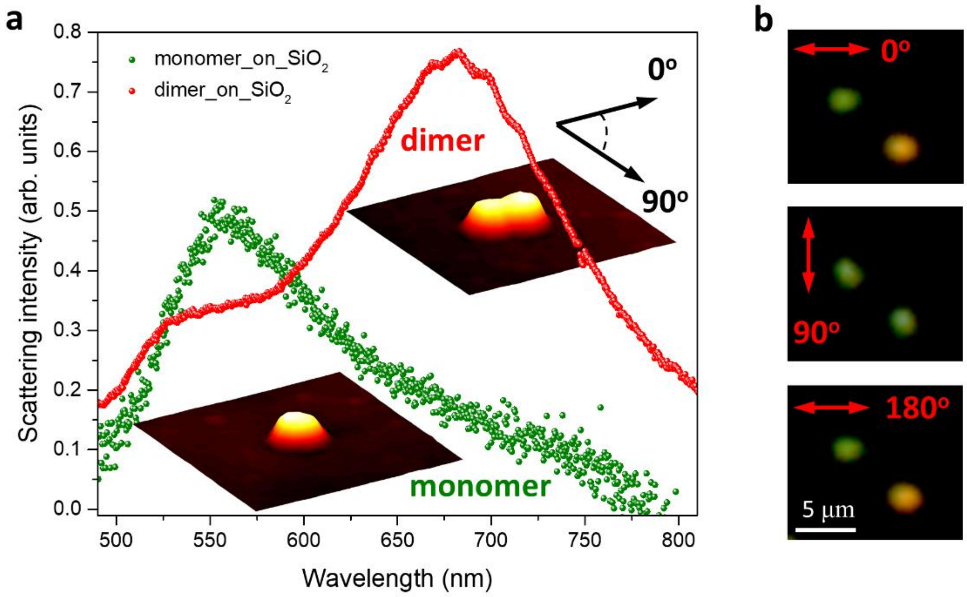

Supplementary Figure 6. a Scattering spectra of a gold nanosphere monomer and dimer on silica. Insets show their morphologies mapped with atomic force microscopy. **b** Scattering patterns of the nanoparticles in (**a**): (always green) monomer and (yellow-green-yellow) dimer. The RND exhibits pronounced polarization dependent scattering intensity pattern, which can be employed to determine its orientation. The double-headed arrows indicate the polarization of the scattered light.

6. Excitation power dependence of the SHG and TPL

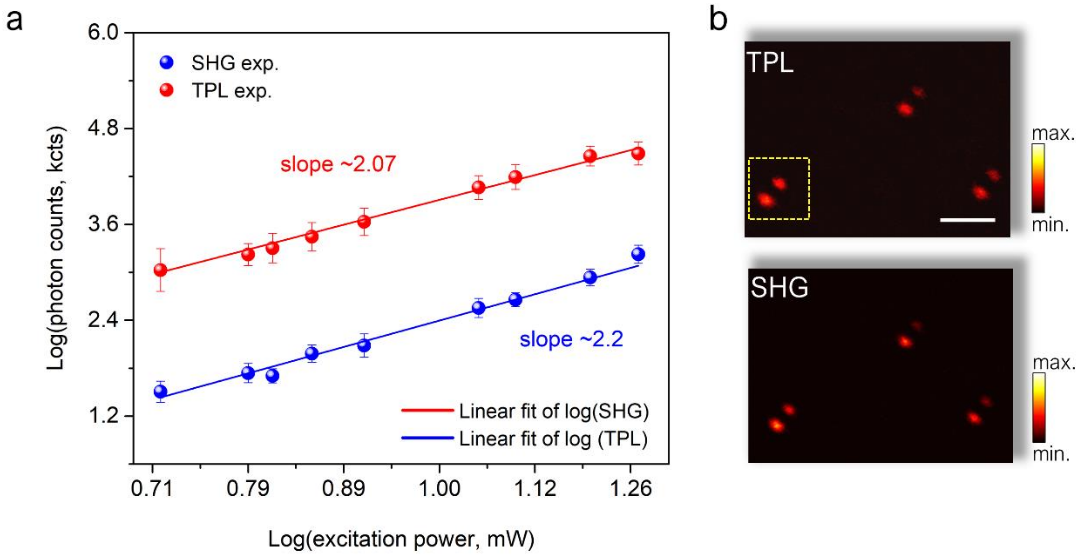

Supplementary Figure 7. a Bi-logarithmic plots of the pump-power dependences of the SHG and the two-photon luminescence (TPL) for a single VND. The quadratic relationship indicates the second-order origin of the second-order harmonic response. **b** TPL and SHG confocal images of the same area containing VNDs. Both TPL and SHG patterns of the individual VNDs exhibit the two-lobe shaped intensity profiles, suggesting these two emission processes are driven by the longitudinal field component. The square-marked nanoparticle corresponds to the one measured in (**a**). The scale bar is 2 μm.

7. Excitation spectra of SHG from single VNDs

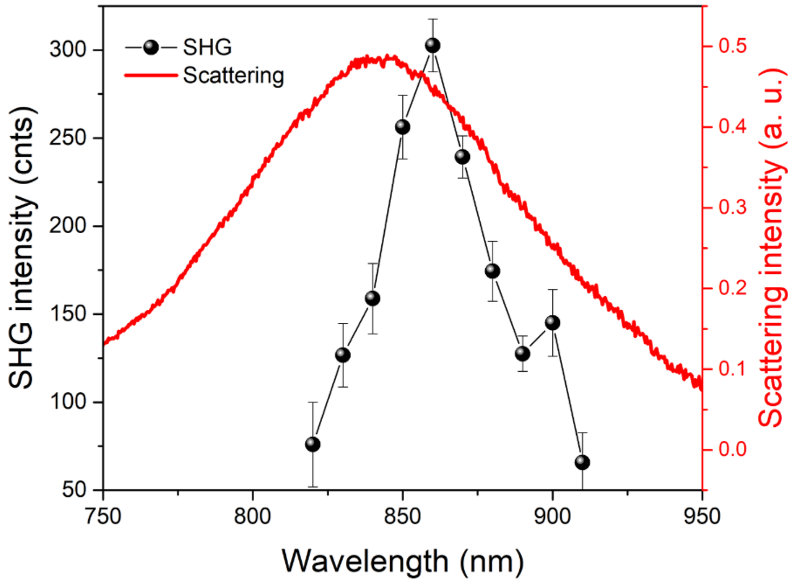

Supplementary Figure 8. The excitation spectrum of the SHG from a single VND. The measured nanostructure has a plasmonic resonance at ~840 nm close to the fundamental wavelength at which strongest SHG is observed. Linear scattering spectrum is shown for comparison. The averaged value for the plot was extracted from 3 measurements with the maximum and minimum values plotted as the error bars.

8. Evaluation of the second harmonic conversion efficiency of metal nanostructures

To evaluate the nonlinear conversion efficiency for VNDs, the signal losses caused by the experimental setup have to be taken into account. The parameters that significantly contribute to the signal attenuation are schematically illustrated in Supplementary Fig. 9a, and their corresponding values are summarized in Supplementary Tables 1-3. The collection efficiency (ζ) of the second harmonic radiation from a single VND is determined by both of the objective NA and the angular SH radiation pattern (Supplementary Fig. 9b). The latter can be derived by performing near-to-far-field transformation of the calculated near-field SHG distribution[11]. The delivery efficiency related to the lens and filter module is considered 100%, which would actually make the SHG conversion efficiency underestimated. The second harmonic signals from the single nanoparticles are extracted from the confocal images (Supplementary Fig. 9d). 271 nanoparticles with comparable SH radiation intensities are selected to give the averaged second harmonic conversion efficiency (Supplementary Fig. 9e). To reduce the noise in the measured signal due to the environmental variations and the possibility of laser damage, the time duration of laser spot dwelling at each pixel is set extremely low, < 20 μs. Even though we are still able to observe the well-resolved SHG patterns of single VNDs (Supplementary Fig. 9d).

Supplementary Table 2. Parameters of the experimental setup.

Parameter	Objective collection efficiency	Objective transmittance	Delivery efficiency (dichroic mirrors, filters, etc.)	Detector quantum efficiency
Symbol	ζ	T_0	T	ρ
Value	0.72	0.85	assumed 1.0	0.35
Total detection efficiency: $\sigma = \zeta * T_0 * T * \rho = 0.2142$				

Supplementary Table 3. Parameters of the excitation laser.

Parameter	Excitation λ (nm)	Laser rep. rate (MHz)	Laser pulse length (fs)	Average excitation power (mW)	Exc. Obj. NA	Focal spot diameter (μm)	Peak excitation power (W)
Symbol	λ	ν	$\boldsymbol{\tau}$	P_ω	NA	$\boldsymbol{\delta}$	P'_ω
Formula						1.22λ / NA	$P_\omega/(\upsilon * \tau)$
Value	850	80	120	0.8	0.95	1.1	83

*The focal spot diameter is evaluated by the size of the Airy spot in the objective focal plane.

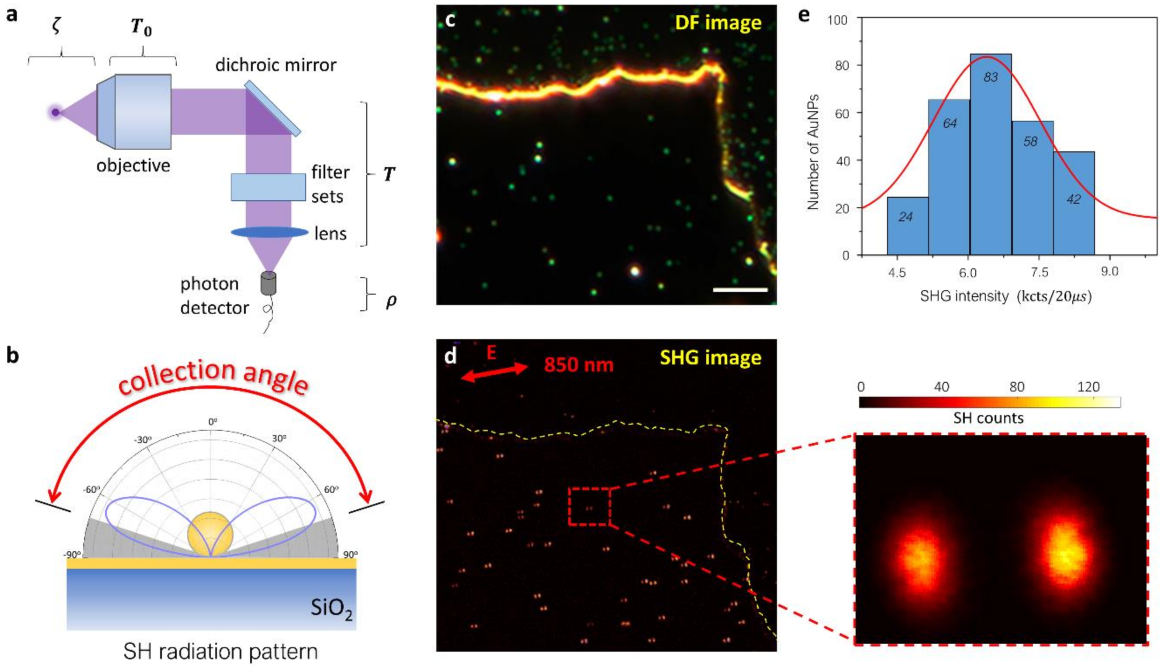

Supplementary Figure 9. a The experimental setup parameters used to evaluate the signal loss, including the collection efficiency ζ, transmittance of the objective T_0, delivery efficiency T associated with the relay optics, and the quantum efficiency (QE) ρ of the detector at the SHG wavelength. **b** Calculated radiation pattern of the SHG from a single VND. The shadowed area (with angle $\theta > 73°$) indicates the angular range within which the SHG radiation cannot be collected by the objective. **c, d** The dark-field image (**c**) and the corresponding SH confocal image (**d**) of the same sample region which contains nanoparticles on both gold film and silica substrate. The inset on the right side shows a magnified SH image of the VND mapped with higher resolution. The double-headed arrow indicates the polarization of the fundamental light. The scale bar is 5 μm. **e** The statistics of SHG intensities based on 271 VNDs with comparable signal amplitudes. The histogram is fitted by a Gaussian function (red curve).

Supplementary Table 4. Analysis of the SHG signal from a single 100-nm-diameter VND.

Parameter	SH emitted photons (cts/s)	SHG power (W)	SHG Peak Power (W)	SHG efficiency	SH conversion efficiency (W^{-1})
Symbol	N	$P_{2\omega}$	$P'_{2\omega}$	η_{SHG}	γ_{SHG}
Formula		$N * E_{ph}$	$P_{2\omega}/(\upsilon * \tau)$	$P_{2\omega}/P_{\omega}$	$P'_{2\omega}/{P'_{\omega}}^2$
Value	1.49×10^9	0.7×10^{-9}	0.73×10^{-4}	0.87×10^{-6}	1.05×10^{-8}

Supplementary Table 5. SHG conversion efficiency from a single 100-nm-diameter VND normalized to the acting field component.

Parameter	Average z-component power (mW)	Peak z-component power (W)	SHG efficiency normalized to z-field component	SH conversion efficiency normalized to z-field component (W^{-1})
Symbol	$P_{\omega,z}$	$P'_{\omega,z}$	$\eta_{SHG,z}$	$\gamma_{SHG,z}$
Formula	$\eta_z * P_\omega$	$P_{\omega,z}/\upsilon\tau$	$P_{2\omega}/P_{\omega,z}$	$P'_{2\omega}/(P'_{\omega,z})^2$
Value	0.14	14.25	5.07×10^{-6}	3.56×10^{-7}

*For details about the power weight of the z-component, η_z, see Supplementary Table 1.

Based on the above parameters and measurement results, a SH conversion efficiency as high as 1.05×10^{-8} is obtained (Supplementary Table 4). Note that only the longitudinal field component is responsible for the observed SH response from VNDs. For the focused Gaussian laser beam used in the experiment, the longitudinal field component takes up a small portion of the total input excitation power (Supplementary Table 1), making the measured SHG conversion efficiency underestimated. In order to obtain a true conversion efficiency, the contribution of the longitudinal component should be taken into account (Supplementary Table 5). The power weight of the longitudinal component depends on the NA of the focusing objective (Supplementary Fig. 4a). Excitation beams with higher weight of a longitudinal field component, such as radially polarized beams and other cylindrical vector beams, will therefore further increase the observed SHG intensity[12].

9. Determination of the SH origin of CTAB-coated gold nanoparticles

The gold colloidal nanoparticles have a single bilayer CTAB coating on the surface, which naturally occurs during the nanoparticle synthesis process. In order to determine if CTAB contributes to the SH response of the nanoparticles, we carried out the control experiments on gold nanoparticles with and without CTAB-coating. Since all chemically synthesized nanoparticles have a coating, the uncoated gold nanospheres were obtained using a laser ablation method[13]. To compare the SHG, we selected the nanospheres with a dimeter of ~160 nm with a broad plasmonic resonance covering the fundamental wavelength (850 nm) used in the main experiments. No significant differences in the observed SHG intensity and spectra from coated and uncoated nanoparticles were observed (Supplementary Fig. 10a). We performed statistical analysis on 11 bare and 23 CTAB-coated nanoparticles (Supplementary Fig. 10b). Only small SH peak intensity fluctuations were observed among them, confirming negligible contribution of the CTAB coating to the SHG from gold nanoparticles. Alternatively, the comparison of the SH origin can be based on the FWHM of the SH spectra[14]: a different

FWHM should be expected if the nonlinear response originates from a metal or a different material next to it. No noticeable difference can be observed for the bare and CTAB-coated nanoparticles (Supplementary Fig. 10a), confirming that the detected SHG signal originates predominantly from the gold nanoparticle. The negligible SH response from CTAB is also consistent with its very weak absorption at the fundamental wavelength (CTAB has an absorption maximum at ~193 nm[15]).

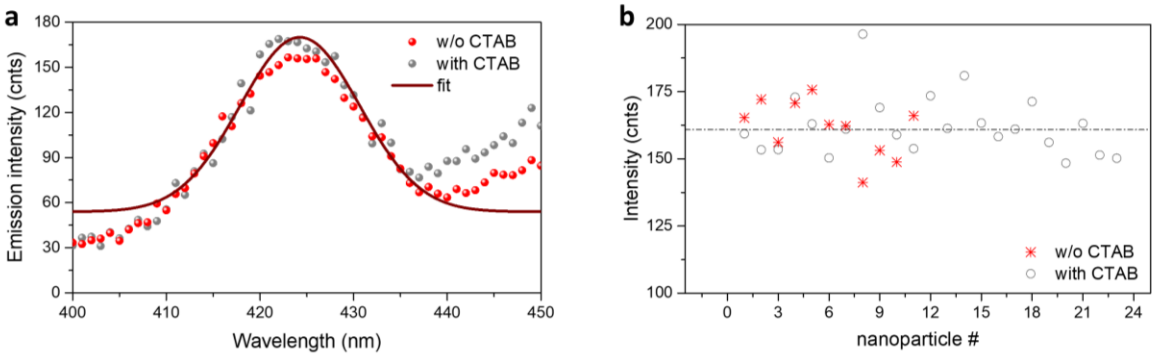

Supplementary Figure 10. a SHG spectra of a ~160 nm gold nanosphere with (gray dots) and without (red dots) CTAB coatings. The solid line is a Lorenz fit of the gray-dot data. **b** Statistics of the SH intensities for 23 CTAB-coated gold nanospheres (circles) and 11 uncoated ones of similar size (stars).

Supplementary References

1. Scalora, M. *et al.* Second- and third-harmonic generation in metal-based structures. *Phys. Rev. A* **82**, 043828 (2010).

2. Krasavin, A. V., Ginzburg, P. & Zayats, A. V. Free-electron optical nonlinearities in plasmonic nanostructures: a review of the hydrodynamic description. *Laser Photon. Rev.* **12**, 1700082 (2018).

3. Marino, G. *et al.* Second-harmonic generation from hyperbolic plasmonic nanorod metamaterial slab. *Laser Photon. Rev.* 12, 1700189 (2018).

4. Ciraci, C. *et al.* Probing the ultimate limits of plasmonic enhancement. *Science* **337**, 1072–1074 (2012).

5. Hajisalem, G., Nezami, M. S. & Gordon, R. Probing the quantum tunneling limit of plasmonic enhancement by third harmonic generation. *Nano Lett.* **14**, 6651–6654 (2014).

6. Zhu, W. *et al.* Quantum mechanical effects in plasmonic structures with subnanometre gaps. *Nat. Commun.* **7**, 11495 (2016).

7. Tame, M. S. *et al.* Quantum plasmonics. *Nat. Phys.* **9**, 329–340 (2013).

8. Bomzon, Z., Gu, M. & Shamir, J. Angular momentum and geometrical phases in tight-focused circularly polarized plane waves. *Appl. Phys. Lett.* **89**, (2006).

9. Novotny, L. & Hecht, B. *Principles of Nano-Optics*, 2nd Ed. (Cambridge University Press, 2012).

10. Born, M. & Wolf, E. *Principles of Optics*. (Pergamon Press, 1980).

11. Yang, J., Hugonin, J. P. & Lalanne, P. Near-to-far field transformations for radiative and guided waves. *ACS Photonics* **3**, 395–402 (2016).

12. Zhan, Q. Cylindrical vector beams: from mathematical concepts to applications. *Adv. Opt. Photonics* **1**, 1 (2009).

13. Chen, J. D. *et al.* Radiation of the high-order plasmonic modes of large gold nanospheres excited by surface plasmon polaritons. *Nanoscale* **10**, 9153–9163 (2018).

14. Aouani, H., Navarro-Cía, M., Rahmani, M. & Maier, S. A. Unveiling the origin of third harmonic generation in hybrid ITO-plasmonic crystals. *Adv. Opt. Mater.* **3**, 1059–1065 (2015).

15. Movchan, T. G., Plotnikova, E. V. & Us'yarov, O. G. Light absorption in solutions of cetyltrimethylammonium and cetylpyridinium bromides. *Colloid J.* **75**, 319–325 (2013).
